# Pollinators in Blackcurrant Orchards: Complementary Roles of Sown and Spontaneous Flower Strips and Limited Spillover of Small Wild Bees Within Fields

**DOI:** 10.1002/ece3.74127

**Published:** 2026-09-10

**Authors:** Mathieu Lachaise, Blanche Collard, Marie‐Charlotte Anstett

**Affiliations:** ^1^ UMR 8079, Écologie Société Évolution Université Paris‐Saclay, CNRS, AgroParisTech Gif‐sur‐Yvette France; ^2^ AgroParisTech Palaiseau Cedex France; ^3^ UMR 6282 Biogéosciences CNRS Université Bourgogne Europe Dijon France

**Keywords:** agri‐environmental scheme, agroecology, bumblebee, floral resources, flower strip, hoverfly

## Abstract

Agricultural intensification threatens pollinators and associated ecosystem services through pesticide use and the loss and fragmentation of natural habitats. Sowing flower strips and preserving spontaneous flowers are commonly recommended practices to support pollinators within fields. However, their effectiveness remains variable across studies and pollinator morphogroups, and their influence on pollinator activity is poorly studied in several crops, particularly in blackcurrant. Here, we studied flowering plant communities (sown and spontaneous) and four pollinator morphogroups (*Bombus*, large wild bee, small wild bee, and hoverfly) in nine blackcurrant orchards in Burgundy (France) during three sampling rounds in spring 2023. A seed mixture was previously sown by farmers in spring 2022 in four orchards, and in autumn 2022 or spring 2023 in the others. In each morphogroup, we investigated whether pollinator abundance in the blackcurrant inter‐rows was influenced by the number of sown and spontaneous flowers and by position within the inter‐rows (edge, inner, centre). We also examined whether pollinator abundance in blackcurrant bushes was related to floral abundance and pollinator activity in adjacent inter‐rows. Flowering plant communities varied across sampling rounds, with sown species flowering only in inter‐rows sown in spring 2022. The abundance of small and large bees increased with the number of spontaneous flowers, whereas hoverfly abundance increased with sown flowers. Small bee abundance, and to a lesser extent that of large bees, decreased in the inner and central transects of the inter‐rows compared to the edge. Moreover, for all morphogroups, pollinator abundance in blackcurrant bushes was not influenced by flower abundance but increased with pollinator abundance in the adjacent inter‐rows. These findings emphasise the complementary value of sown and spontaneous floral resources to support pollinators within orchards. The sharp decline in small bee abundance toward orchard interiors suggests potential dispersal limitations and highlights the importance of integrating suitable habitats to facilitate pollinator movement within crops.

## Graphical abstract

Sowing flower strips and preserving spontaneous flowers are commonly recommended to support pollinators in agricultural landscapes. In nine blackcurrant orchards, these resources played complementary roles: the abundance of small and large wild bees was positively associated with spontaneous flowers, whereas hoverfly abundance was positively associated with sown flowers. Small bee abundance strongly declined toward orchard interiors, suggesting potential dispersal constraints within crops. These results highlight the importance of sown but also spontaneous floral resources to support pollinator communities and facilitate their movement within crops.

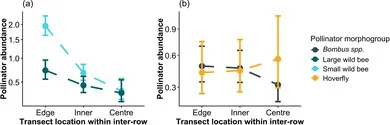

## 1 Introduction

Agricultural intensification, which is associated with the loss and fragmentation of natural habitats as well as the increasing use of insecticides worldwide (FAO 2024), is widely recognised as a major driver of insect pollinator decline (Potts et al. 2010; Kennedy et al. 2013; Goulson et al. 2015). This decline threatens pollination services, a crucial ecological process that sustains both natural and agricultural ecosystems (Klein et al. 2007; Vanbergen et al. 2013; Potts et al. 2016). Indeed, approximately 90% of flowering plants are animal‐pollinated (Ollerton et al. 2011), while 28%–61% of crops worldwide are currently pollinator‐limited (Turo et al. 2024). Pollination therefore directly influences food production, quality, and security for humans, thereby supporting human well‐being (Potts et al. 2016; Katumo et al. 2022).

In this context, bees and hoverflies are essential pollinators (Klein et al. 2007; Doyle et al. 2020). These species exhibit diverse ecological requirements (Doyle et al. 2020; Tschanz et al. 2023) and may visit distinct flower assemblages, whether sown or spontaneous (Kleijn et al. 2015; Junqueira et al. 2022). This functional diversity enhances the spatial and temporal complementarity of pollination and is therefore crucial for maintaining the productivity and stability of natural and agricultural ecosystems (Klein et al. 2007; Junqueira et al. 2022), as well as the resilience of pollination networks (Bascompte and Scheffer 2023). Amid agricultural intensification, the persistence of pollinator communities in agricultural landscapes depends on the availability of suitable habitats such as nesting sites, as well as floral resources that provide nectar and pollen throughout the season (Scheper et al. 2013; Goulson et al. 2015).

Increasing floral resources in agricultural fields, by sowing flower strips or maintaining spontaneous flowers, has been widely promoted to support wild pollinator communities and associated pollination services (Campbell et al. 2017; Knapp et al. 2019; Albrecht et al. 2020). Such within‐field floral resources may also partly compensate for limited pollinator spillover from surrounding habitats into crop interiors (Ganser et al. 2018; MacInnis et al. 2020). Although many studies have demonstrated the benefits of these measures on pollinator communities (Feltham et al. 2015; Buhk et al. 2018; Knapp et al. 2019), their effectiveness remains variable, depending on flower composition and pollinator taxa considered (Scheper et al. 2021), time since implementation (Lowe et al. 2021; Albrecht et al. 2021; Schmied et al. 2022), and landscape context (Haenke et al. 2009; Scheper et al. 2021). In addition, the expected increase in crop pollination is not always observed (Campbell et al. 2017; Albrecht et al. 2020; Azpiazu et al. 2020). These inconsistencies in pollination benefits may depend on pollinator identity and behaviour (e.g., floral preferences) and result from competition for visits between crops and non‐crop flowers, whether spontaneous or sown (Desaegher et al. 2021; Carturan et al. 2023).

While increasing local flowering resources can benefit pollinator communities, their abundance may also be influenced by landscape composition, such as the surface of (non‐)flowering crops, semi‐natural habitats, and urban areas (Le Féon et al. 2013; Senapathi et al. 2017; Krimmer et al. 2019; Bottero et al. 2023; Neira et al. 2024). In addition, pollinator communities fluctuate over time due to seasonal dynamics (Rohde and Pilliod 2021; Encinas‐Viso et al. 2023; Schulte et al. 2025), and agricultural landscapes change with the blooming of mass‐flowering crops and the dynamics of spontaneous floral communities within and around fields (Eeraerts et al. 2021; Maurer et al. 2022; Ammann et al. 2024).

The mass‐flowering blackcurrant crops ( *Ribes nigrum* L.), particularly the traditional *Noir de Bourgogne* variety, are highly dependent on bee pollination for fruit production (Anstett et al. 2019). Following modern selection in the 1980s, only two out of four remnant clones of this variety remain, co‐planted with 15%–20% of a pollen donor variety, typically *Royal de Naples*, to ensure cross‐pollination (Anstett et al. 2019). Blackcurrants bloom in early spring, and their small, bell‐shaped flowers have a relatively narrow corolla, restricting nectar access for bees with short proboscises (Mignot 2023). Main visitors are *Apis mellifera* , medium‐ to large‐sized *Andrena* spp., *Bombus* spp., and managed *Osmia bicornis* (Anstett et al. 2019; Mignot 2023). However, *Bombus* spp. and *Apis mellifera* are not faithful pollinators of blackcurrant, as they prefer more accessible crop resources (e.g., rapeseed, mustard, cherry). Faced with a sharp decline in blackcurrant yields since 2010, farmers are currently developing an agroecological system in which blackcurrant pollination is supported by *Osmia bicornis* reared on the farm, which has resulted in a 20%–40% increase in blackcurrant yield within an 80 m radius of these nesting boxes (Anstett et al. 2022). In addition, farmers enhanced floral resources in orchard inter‐rows by reducing mowing and by sowing a seed mixture of local plant species to support wild pollinator communities. However, the benefits of such practices for pollinator communities in inter‐rows have, to our knowledge, not yet been studied in blackcurrant orchards, and it remains unclear whether greater pollinator activity in these inter‐rows translates into increased pollinator activity in adjacent blackcurrant bushes.

In this study, we surveyed four morphogroups of floral visitors (*Bombus* spp., large wild bees, small wild bees, and hoverflies) in nine blackcurrant orchards in Burgundy (France) during three sampling rounds in spring 2023. Hereafter, we refer to these morphogroups as pollinators, given the strong evidence that many species within these taxa contribute to the pollination of numerous flowering plant species in agricultural and natural ecosystems (Potts et al. 2016; Doyle et al. 2020; Junqueira et al. 2022). We also surveyed spontaneous and sown flower communities. We then analysed: (1) how sown and spontaneous flower communities varied across sampling rounds and sowing dates, and (2) how the abundance of each pollinator morphogroup in the inter‐rows was influenced by floral resource abundance and transect location along the inter‐row (edge, inner, centre), accounting for landscape composition. We then further investigated (3) whether higher abundance of each pollinator morphogroup in these inter‐rows translated into higher abundance of the same morphogroup in adjacent blackcurrant bushes. We expected sown and spontaneous flower communities to vary rapidly between sampling rounds, with flowers sown in spring and autumn 2022 being more abundant than those sown in spring 2023. We hypothesised that, for each pollinator morphogroup, abundance in the inter‐rows would be positively related to the abundance of sown and spontaneous floral resources. We further expected pollinator abundance to be lower in the centre and inner parts of inter‐rows than at their edges. Finally, we expected pollinator abundance in blackcurrant bushes to increase with pollinator abundance of the same morphogroup in adjacent inter‐rows.

## 2 Material and Methods

### 2.1 Study Sites

We collected data from nine blackcurrant orchards (hereafter referred to as sites, Figure S1) managed by farmers participating in the pollinator restoration project in Burgundy (France). The orchards were located across a gradient of landscape compositions, from intensive agricultural areas dominated by large fields to semi‐natural and artificial landscapes with a lower proportion of crops (Figure S1). Orchard size ranged from 1.6 to 13.6 ha, with blackcurrant bushes ( *Ribes nigrum* varieties: *Noir de Bourgogne*, *Royal de Naples*, and *Andorine*) of varying ages (3–13 years) planted in rows spaced 3 m apart. The minimum distance between orchards ranged from 720 to 18,220 m (mean = 4980 ± 7525 m).

In each orchard, farmers used two approaches to supplement their blackcurrant inter‐rows with floral resources: by (1) preserving spontaneous flowers, and by (2) sowing flowers in one inter‐row. Flowers were sown in spring 2022 in four orchards and in autumn 2022 or spring 2023 in the remaining five orchards. The seed mixture sown for the flower strip (‘mélange Cassis’, supplied by Nungesser) consisted of 20% local wildflowers (which may occur spontaneously in Burgundy) to provide resources for pollinators, and 80% local wild grass species selected for their low size and competitiveness. Details of the seed mixture composition are provided in Table S1. The flowering species were selected for their varied phenologies to provide resources for wild pollinators from the peak of blackcurrant flowering until September. To support pollination before the restoration of wild pollinator populations, farmers also placed 700 *Osmia bicornis* cocoons and one nesting box in the middle of the sown flower strips.

### 2.2 Sampling Design Implemented in Blackcurrant Orchards

**FIGURE 1:**
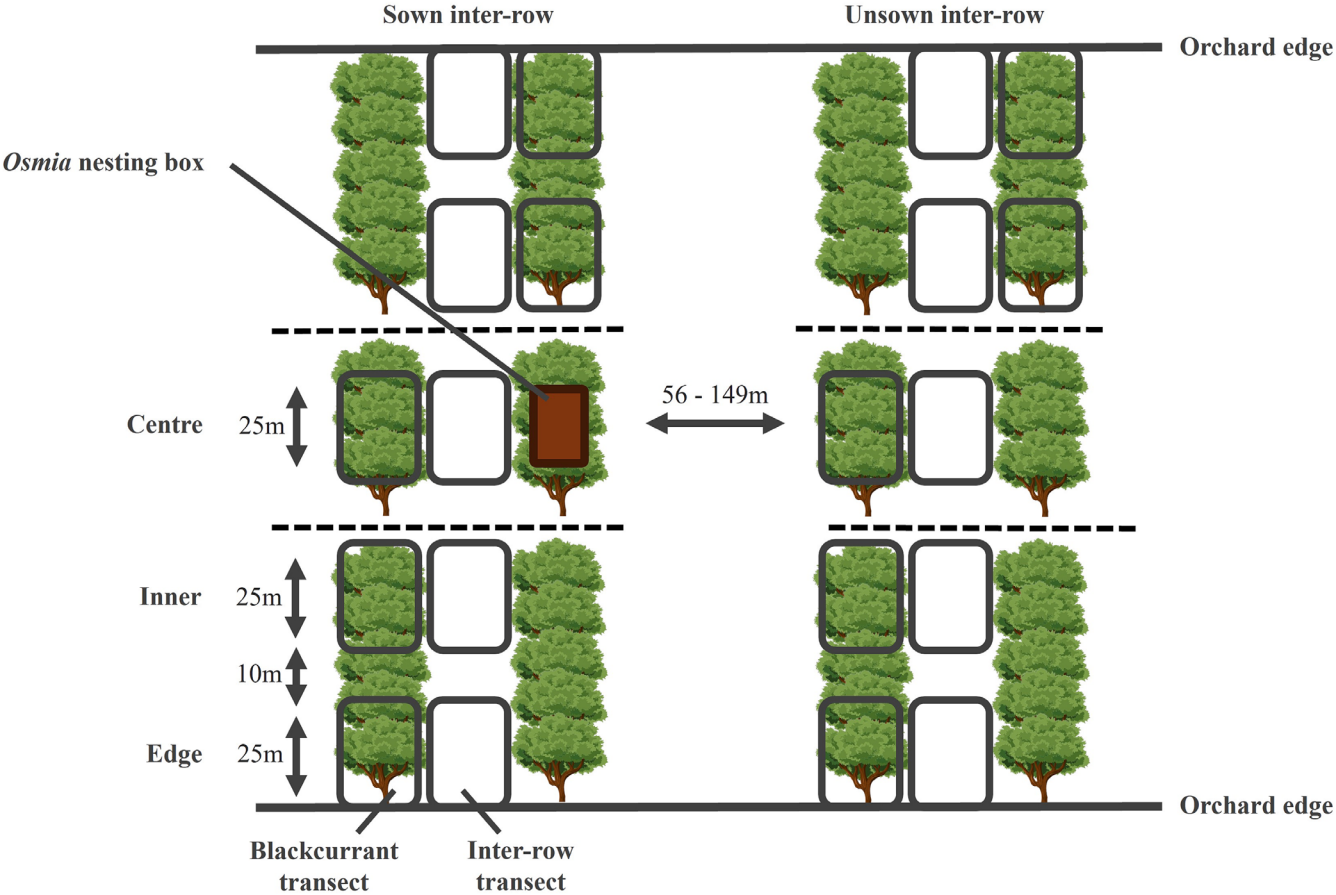
Experimental setup implemented in the nine blackcurrant orchards. The black rectangles represent the transects where surveys were conducted, both in sown and unsown inter‐rows (inter‐row transects) and in adjacent blackcurrant rows (blackcurrant transects): Edge (0–25 m), inner (35–60 m), and centre (69–238 m, depending on plot size). No centre transects were performed at Site 1 and Site 7, which were too small in length.

In each orchard, we delimited five transects (25 m × 1–1.5 m) in which we surveyed pollinators and flowering plants in the sown inter‐row (inter‐row transect; Figure 1), and five transects (25 m × 1–1.5 m) where we surveyed pollinators in the adjacent blackcurrant rows (blackcurrant transect). Four transects were located at opposite edges of the inter‐row (0–25 m), four further inside (35–60 m), and two at the centre of the inter‐row (69–238 m, depending on plot size). We then replicated the same design (inter‐row and blackcurrant transect) in an unsown inter‐row with spontaneous flowers (Figure 1), as far as possible from the sown inter‐row (56–149 m, depending on orchard configuration).

### 2.3 Plant and Pollinator Surveys

Data collection occurred during the blackcurrant flowering period between 19 Apr 2023 and 12 May 2023, under suitable conditions for pollinator activity: between 10:00 am and 4:00 pm on sunny or brightly overcast days with temperatures above 13°C. By visiting the fields on every suitable day, we managed to sample each orchard on three different dates (sampling rounds) across the blackcurrant flowering period: sampling round 1 from 19 April 2023 to 28 April 2023, sampling round 2 from 28 April 2023 to 4 May 2023 (except for site 2 that was sampled on 10 May 2023 due to meteorological constraints), sampling round 3 from 5 May 2023 to 12 May 2023. The mean interval between two consecutive sampling rounds was 6.83 ± 2.85 days across sites.

#### 2.3.1 Flowering Plant Survey

On each sampling round, we counted the number of floral units of all flowering species (excluding Poaceae) in all sown and unsown inter‐row transects, resulting in a total of 86 transects per sampling round across the study. A floral unit was defined as an individual flower or inflorescence that required small pollinators to take flight to move to another unit (Baldock et al. 2015). Specifically, inflorescences in capitula (e.g., Asteraceae) and spike‐shaped inflorescences (e.g., *Onobrychis viciifolia* ) were considered single floral units. Plants were identified to the species level, or to the genus level in cases of uncertainty (e.g., *Ranunculus* spp., *Taraxacum* spp., *Veronica* spp.). For species not included in our seed mixture, we used the PlantNet and iNaturalist applications to confirm identification and ensure that they had been previously observed in the region. We distinguished plant taxa included in the seed mixture (sown flowers) from the others (spontaneous flowers). The seed mixture contained wildflower species that can also occur spontaneously in Burgundy. However, only 19 out of 16,106 floral units from four species included in the seed mixture were recorded in unsown transects, suggesting that this case was limited: *Anthyllis vulneraria* (*n* = 1), *Leucanthemum vulgare* (*n* = 1), *Sanguisorba minor* (*n* = 16), *Trifolium incarnatum* (*n* = 1). These plants could be either spontaneous or re‐sown from the floral strip. The flowering plants identified to genus or species level and observed in sown and unsown transects are provided in Table S2.

#### 2.3.2 Pollinator Survey

Pollinators flying, resting on leaves, and foraging on flowers were recorded by slowly walking along each inter‐row and blackcurrant transect for 1 min, in both sown and unsown inter‐rows (Figure 1), resulting in a total of 516 transects (516 min of observation). Depending on the orchard exposure, we selected the adjacent blackcurrant rows to the right or left of the inter‐row spaces to minimise the potential effects of sun exposure. All observations were conducted by the same person (ML). Pollinators were classified into nine easily identifiable morphogroups: *Apis mellifera* , *Osmia bicornis* , *Bombus* spp., large wild bees (> 10 mm), small wild bees (< 10 mm), kleptoparasitic bees, hoverflies, bee flies and butterflies.

### 2.4 Characterisation of the Landscape Composition

To account for variation in landscape context between and within orchards, we evaluated landscape composition within a 100 m buffer around the middle point of each pair of inter‐row and adjacent blackcurrant transect through photo‐interpretation of the 2023 Google Satellite imagery layer in QGIS (version 3.34, Prizren), followed by field verification (Figure S1, which includes detailed maps of the landscape around the nine orchards at a 500 m radius). This approach enabled us to identify four main landscape categories likely to influence flower‐visiting insects (Table S3): natural and semi‐natural habitats (e.g., woods, hedgerows, grasslands, aquatic environments), artificial areas (e.g., hardened surfaces and dwellings), flowering crops (in bloom during our study) and non‐flowering crops (not in bloom during our study, also including all cereal fields).

### 2.5 Data Analysis

#### 2.5.1 Variation in Flowering Plant Communities Across Transects and Sampling Rounds

All data analyses were performed in R (R Core Team 2023 version 4.3.2).

To evaluate the variation in flower communities between transects and sampling rounds, we first calculated the total number of floral units in each sown and spontaneous inter‐row transect sampled. As plant communities may shift rapidly across the season, we removed from this analysis the data from Site 2 in sampling round 2 (10 transects), which were sampled later in comparison to other sites (see section ‘2.3 Plant and pollinator surveys’). For each sampling round, we then computed a Bray–Curtis dissimilarity matrix from floral unit abundances using the *vegdist()* function in the vegan package (Oksanen et al. 2022; version 2.6‐4), excluding taxa occurring in only a single transect (singletons) to reduce noise from rare taxa. A principal coordinates analysis (PCoA) with two dimensions was then performed based on each dissimilarity matrix using the *wcmdscale()* function in vegan, and flowering taxa were fitted to the axes using the *envfit()* function in vegan with 999 permutations to evaluate their relationship with the ordination.

#### 2.5.2 Effect of the Local and Landscape Factors on Pollinator Abundance in the Inter‐Rows

To evaluate how pollinator abundance varied with the abundance of the sown and spontaneous flowers, as well as transect position within the inter‐rows (edge, inner, and centre), we restricted this analysis to the inter‐row transects (*n* = 258). Inter‐row transects were preferred over blackcurrant transects, as they were expected to provide higher pollinator detection rates and more accurate estimates of their actual abundance in each transect. As the abundance of butterflies (38 individuals), kleptoparasitic bees (7), and bee flies (1) was very low, these groups were excluded from the analysis. We also removed *Osmia bicornis* and *Apis mellifera*, whose abundance might not fully reflect wild populations, as it could have been influenced by the presence of nesting boxes within orchards and nearby beehives, respectively. We therefore focused our analyses on four morphogroups: *Bombus* spp., large wild bees, small wild bees, and hoverflies.

For each morphogroup, we fitted a generalised linear mixed model (GLMM) with local (inter‐row position and flower abundance) and landscape variables presented in Table 1 (see Figure S2 for correlation plot), as well as the sampling round to account for temporal variability, using the *glmmTMB()* function from the *glmmTMB* package (Brooks et al. 2017; version 1.1.8). We used a Poisson distribution with a log link for all models. Although pollinators may respond to landscape context at spatial scales larger than 100 m (Steffan‐Dewenter et al. 2002; Westphal et al. 2003; Le Féon et al. 2013), our sampling design did not allow us to test such effects. We therefore included Site (*n* = 9) as a random factor to account, in part, for large‐scale landscape variation among orchards. We then performed model selection using the *dredge()* function from the *MuMIn* package (Bartoń 2024; version 1.48.4) to identify the combination of variables that minimised the Akaike information criterion (AIC). All quantitative predictors were standardised using the *scale()* function.

**TABLE 1:** Description of the explanatory variables used in the models. For each quantitative variable, the mean, standard deviation (S.D.), minimum (Min.) and maximum (Max.) values are presented.

Type	Variable	Description	Mean (± S.D.)	Min.	Max.
**Response variables**					
Pollinator abundance	*Bombus* spp. abundance	Number of *Bombus* spp. individuals per transect	0.49 (±0.76)	0	4
	Large wild bee abundance	Number of large wild bee individuals per transect	0.52 (±0.84)	0	6
	Small wild bee abundance	Number of small wild bee individuals per transect	1.17 (±1.41)	0	11
	Hoverfly abundance	Number of hoverfly individuals per transect	0.68 (±1.38)	0	12
**Explanatory variables**					
Local variables	Inter‐row position	Position of transect within the blackcurrant inter‐row and the associated adjacent blackcurrant row (Edge, Inner, Centre)	—	—	—
	Spontaneous flower abundance	Number of floral units from spontaneous plants in each transect	55 (±148)	0	1405
	Sown flower abundance	Number of floral units from sown plants in each transect	62 (±237)	0	2022
Landscape variables	Semi‐natural habitat surface (100 m)	Total area of woods, hedgerows, grasslands and aquatic environments (m^2^)	6352 (±5049)	0	20,813
	Artificial area surface (100 m)	Total area of dwellings, concrete surfaces and anthropogenic mineral surfaces (m^2^)	921 (±1639)	0	10,714
	Flowering crop surface (100 m)	Total area of crops in bloom during our study (m^2^)	19,589 (±5490)	9179	31,402
	Non‐flowering crop surface (100 m)	Total area of crops not in bloom during our study (m^2^)	4544 (±5464)	0	19,614
Temporal variable	Sampling round	Round number in which pollinators and flowers were sampled in each orchard (1, 2, 3)	—	—	—
Random factor	Site	Sites (*n* = 9) where pollinators and flowers were surveyed	—	—	—

#### 2.5.3 Influence of Flowers and Pollinators in Inter‐Rows on Pollinators in Blackcurrant Bushes

After evaluating the factors influencing the abundance of each pollinator morphogroup in the inter‐row transects, we investigated whether a higher abundance of each morphogroup in these transects also translated into a higher abundance of the same morphogroup in the adjacent blackcurrant transects. We focused on the same morphogroups as in the previous analysis: *Bombus* spp., large wild bees, small wild bees, and hoverflies. For each pollinator morphogroup, we fitted a GLMM using *glmmTMB()*, with pollinator abundance in blackcurrant transects as the response variable and the abundance of the same pollinator morphogroup in the adjacent inter‐row transects as the main explanatory variable. We also included the abundance of sown and spontaneous flowers to investigate if their presence influenced pollinator activity in blackcurrant bushes, as well as the inter‐row position and the landscape and temporal variables (Table 1) to account for spatial and temporal context. Models were fitted with a Poisson error distribution and a log link, including *Site* (*n* = 9) as a random factor. We then performed model selection using the *dredge()* function. All quantitative predictors were scaled using the *scale()* function.

#### 2.5.4 Model Evaluation and Graphical Representation

We tested for residual overdispersion and zero inflation using the T*estDispersion()* and *testZeroInflation()* functions in the *DHARMa* package (Hartig 2022; version 0.4.6). No evidence of overdispersion or zero inflation was detected. We checked for multicollinearity by calculating the variance inflation factors (VIFs) using the *check_collinearity()* function from the *Performance* package (Lüdecke et al. 2021; version 0.12.4). All VIF's were below 2, which is considered acceptable (Zuur et al. 2010). For several models (see Table 2 and Table S5), we detected spatial autocorrelation in the residuals using Moran's I, computed with the *testSpatialAutocorrelation()* function from the *DHARMa* package. In such cases, we fitted spatial GLMMs using the *spaMM* package (Rousset and Ferdy 2014; version 4.5.0), which allows the inclusion of a Matérn term to account for transect spatial coordinates. For all models on pollinator abundance, we tested excluding data from Site 2 in sampling round 2, which was sampled later than the other sites, and it did not affect the results presented below.

Figures were produced using the *ggplot2* package (Wickham 2016). Regression lines represent model predictions obtained using the *ggpredict()* function from the *ggeffects* package (Lüdecke 2018).

## 3 Results

### 3.1 Pollinator and Flowering Plant Communities Observed in Blackcurrant Orchards

A total of 2004 pollinators were observed during our survey, corresponding to approximately 4 individuals per minute: small wild bees (606 individuals), *Apis mellifera* (408), hoverflies (352), large wild bees (270), *Bombus* spp. (251), *Osmia bicornis* (71), butterflies (38), kleptoparasitic bees (7) and bee flies (1). We identified 46% of these individuals to the genus level: *Apis* (408), *Bombus* (251), *Andrena* (191), *Osmia* (71), *Halictus* (15), *Anthophora* (7), *Lasioglossum* (7), *Nomada* (5), *Sphecodes* (2), *Colletes* (1) and *Eucera* (1) (Table S4). We only observed seven *Andrena fulva* , which was comparable in abundance to *Bombus* spp. and *Apis mellifera* in an unpublished pollinator census of blackcurrant orchards in 1981 (Anstett et al. 2019).

Overall, we recorded 30,194 floral units across all transects (Table S2): 16,106 (53%) were from the species of the seed mixture sown in the inter‐rows, whereas 14,088 (47%) developed spontaneously. Among the sown species, the most abundant were *Sanguisorba minor* (14,128 floral units), *Borago officinalis* (1048), *Leucanthemum vulgare* (413), and *Trifolium incarnatum* (400). The most abundant spontaneous taxa were *Taraxacum* spp. (8215), *Lamium purpureum* (1359), *Ranunculus* spp. (1161) and *Matricaria* spp. (559). While flower species included in the seed mixture were observed almost exclusively in the sown inter‐rows (16,087 out of 16,106), spontaneous flowers were detected in both the sown (4269) and unsown (9819) inter‐rows.

### 3.2 Variation in Flowering Plant Communities Across Transects and Sampling Rounds

**FIGURE 2:**
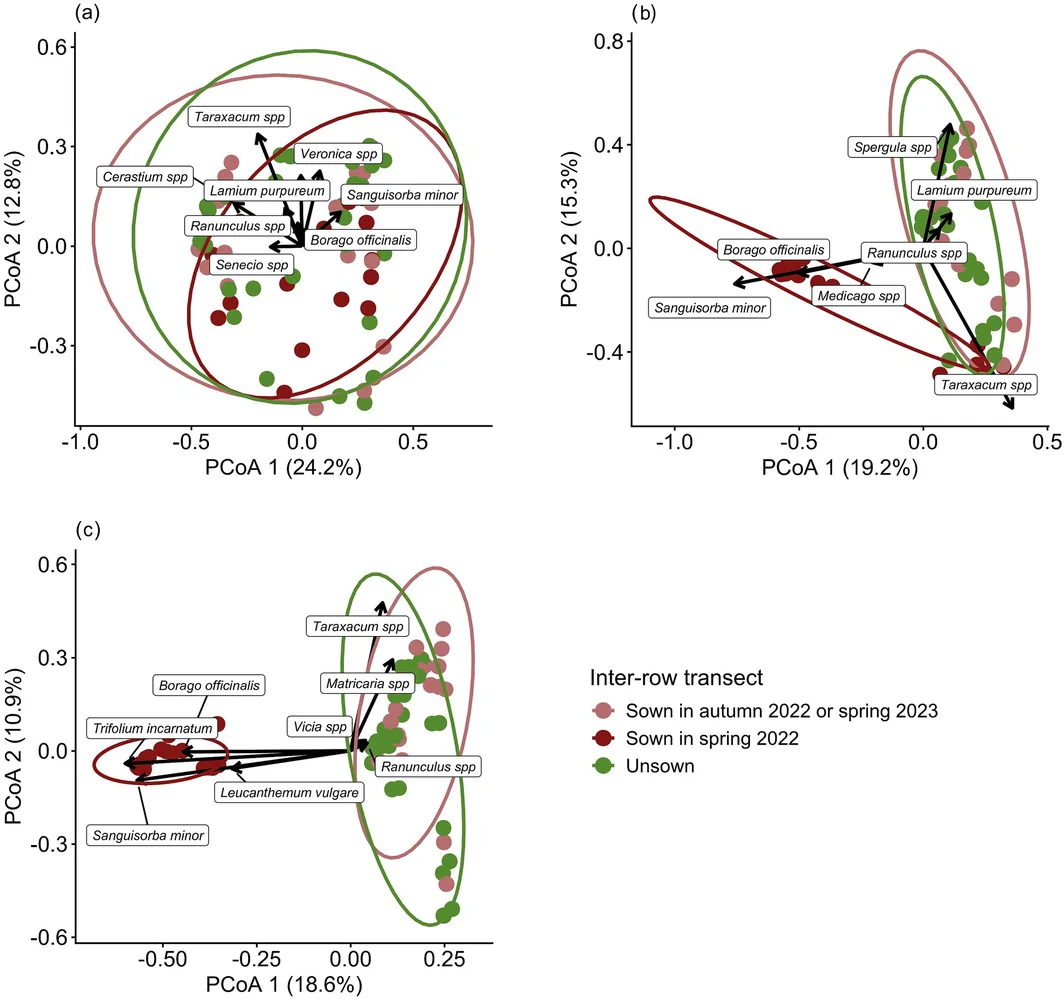
Principal coordinates analysis (PCoA) of floral community composition in unsown (green) and sown transects (spring 2022 in dark red and autumn 2022‐spring 2023 in light red) during the (a) first, (b) second, and (c) third sampling round. Each point represents a transect (*n* = 86 in round 1 and 3; *n* = 76 in round 2). Only the eight most abundant flowering species at each round are shown. Arrows indicate fitted species vectors, showing the direction and strength of their association with community composition.

During the first sampling round, the composition of flowering communities was similar among unsown and sown inter‐rows, independently of the sowing date (spring 2023 vs. autumn 2022 and spring 2022; Figure 2a). However, during the second sampling round, and more markedly the third one, flowering communities in the inter‐rows sown in spring 2022 became clearly distinct from other inter‐rows in the ordination space (Figure 2b,c). This shift was partly associated with the flowering of the sown species *Sanguisorba minor* and *Borago officinalis* in sampling round 2, with the addition of *Leucanthemum vulgare* and *Trifolium incarnatum* in sampling round 3. In contrast, inter‐rows sown later (October 2022 and spring 2023) exhibited floral compositions that were relatively similar to unsown inter‐rows in all sampling rounds (Figure 2).

### 3.3 Effect of Local and Landscape Factors on Pollinator Abundance Varies Between Morphogroups and Sampling Rounds

**FIGURE 3:**
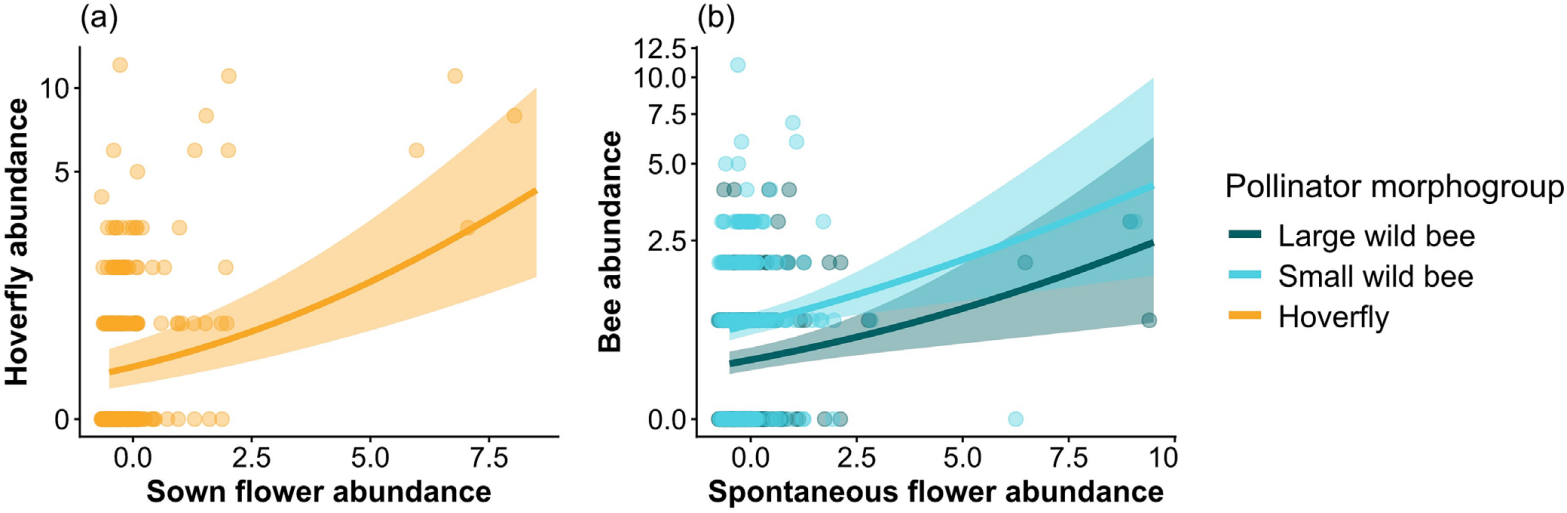
(a) Hoverfly abundance (black line) in relation to the number of sown flowers, and (b) large (dark blue) and small (light blue) wild bee abundance in relation to the number of spontaneous flowers. Solid lines show model predictions, while shaded areas represent 95% confidence intervals. See Table 2 for model details. Y‐axes are log_10_(*y* + 1) transformed, and explanatory variables (sown flower abundance and spontaneous flower abundance) are scaled.

Pollinator morphogroups responded differently to the number of sown and spontaneous flowers, as well as to transect position within the inter‐rows. The abundance of hoverfly increased significantly with the number of sown flowers (*E* = 0.26 ± 0.05, *p* < 0.001; Figure 3a and Table 2), whereas the abundance of small and large bees increased significantly with the number of spontaneous flowers (small bees: *E* = 0.16 ± 0.05, *p* = 0.001; large bees: *E* = 0.16 ± 0.05, *p* < 0.001; Figure 3b and Table 2). These effects were in part driven by a high number of sown flowers in four transects (Figure 3a) and spontaneous flowers in three transects (Figure 3b). However, removing these points did not change the results, except for small wild bees, for which a slight positive trend was observed (*p* = 0.065). In contrast, the abundance of *Bombus* spp. did not vary with the abundance of flowers, whether sown or spontaneous (Table 2).

**FIGURE 4:**
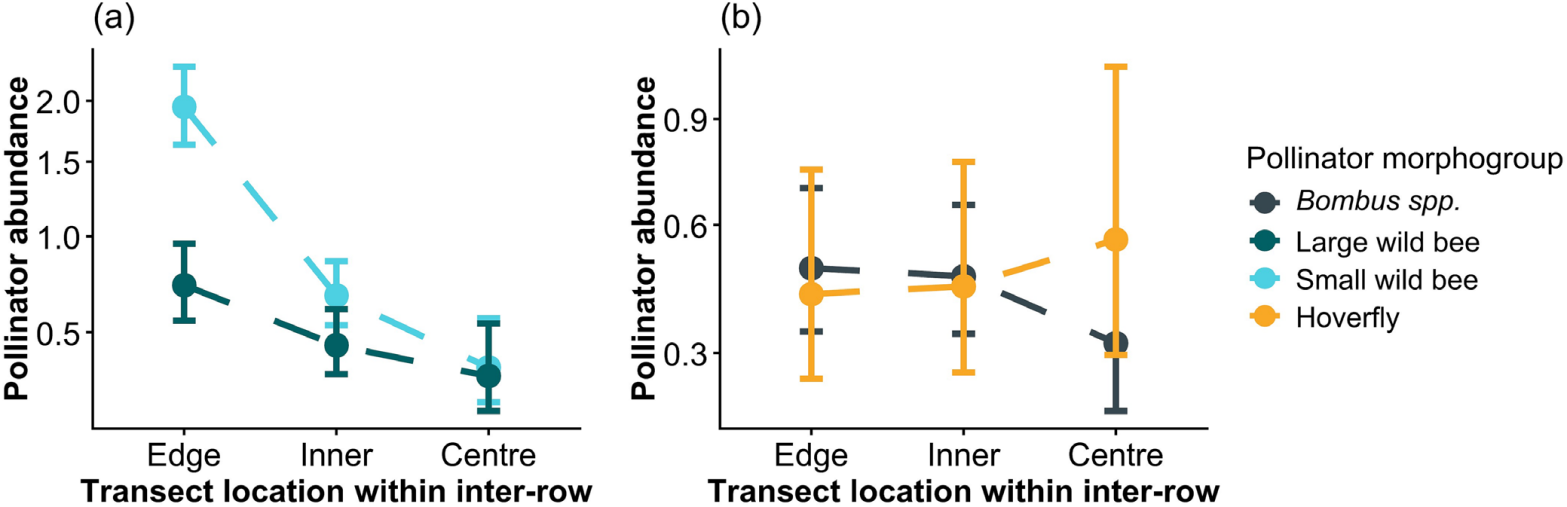
Predicted abundance of (a) small and large wild bees, and (b) Bombus spp. and hoverflies in the edge, inner, and centre inter‐row transects. Solid points show model predictions, while vertical lines represent 95% confidence intervals. See Table 2 for model details. For Bombus spp. and hoverflies, full models with all variables were used, as the transect variable was not selected in the models with the lowest AIC. Y‐axes are log_10_(*y* + 1) transformed.

The abundance of small bees, and to a lesser extent large bees, decreased significantly from the edge to the inner inter‐row transect (small bees: *E* = −1.06 ± 0.14, *p* < 0.001; large bees: *E* = −0.50 ± 0.18, *p* = 0.006; Figure 4a and Table 2), and further to the centre inter‐row transect (small bees: *E* = −1.71 ± 0.26, *p* < 0.001; large bees: *E* = −0.83 ± 0.29, *p* = 0.004; Figure 4a and Table 2). The abundance of *Bombus* spp. did not vary with flower abundance and, similarly to hoverflies, showed no significant variation among edge, inner, and centre inter‐row transects (Figure 4b).

**TABLE 2:** Final models selected based on Akaike information criterion for *Bombus* spp., large wild bees, small wild bees and hoverflies. ‘*n*’ indicates the total number of pollinators counted for each morphogroup. Trigamma *R* ^2^ *c* and *R* ^2^ *m* values are reported (Nakagawa et al. 2017). Spatial models were performed for *Bombus* spp. and hoverflies due to spatial autocorrelation in residuals.

Response variable	Explanatory variables	Estimate (±SE)	*z* value	*p*
*Bombus* spp. abundance *n* = 133 *R* ^2^ *m* = 0.03 *R* ^2^ *c* = 0.05	Artificial area surface (100 m)	−0.40 (± 0.17)	−2.30	0.021*
Large wild bee abundance *n* = 150 *R* ^2^ *m* = 0.04 *R* ^2^ *c* = 0.05	Spontaneous flower abundance	0.16 (± 0.05)	3.30	< 0.001***
	Edge—Inner	−0.50 (± 0.18)	−2.74	0.006**
	Edge − Centre	−0.83 (± 0.29)	−2.89	0.004**
	Non‐flowering crop surface (100 m)	−0.30 (± 0.11)	−2.72	0.007**
	Artificial area surface (100 m)	−0.15 (± 0.10)	−1.50	0.135
Small wild bee abundance *n* = 298 *R* ^2^ *m* = 0.23 *R* ^2^ *c* = 0.24	Spontaneous flower abundance	0.16 (± 0.05)	3.28	0.001**
	Sown flower abundance	0.09 (± 0.06)	1.70	0.090⸱
	Edge − Inner	−1.06 (± 0.14)	−7.45	< 0.001***
	Edge − Centre	−1.71 (± 0.26)	−6.55	< 0.001***
	Sampling round 1 − Sampling round 2	0.48 (± 0.16)	3.10	0.002**
	Sampling round 1 − Sampling round 3	0.44 (± 0.16)	2.74	0.006**
	Flowering crop surface (100 m)	0.18 (± 0.08)	2.28	0.023*
	Non‐flowering crop surface (100 m)	−0.22 (± 0.08)	−2.90	0.004**
Hoverfly abundance *n* = 210 *R* ^2^ *m* = 0.20 *R* ^2^ *c* = 0.35	Sown flower abundance	0.26 (± 0.05)	4.96	< 0.001***
	Sampling round 1 − Sampling round 2	0.59 (± 0.27)	2.19	0.029*
	Sampling round 1 − Sampling round 3	1.66 (± 0.24)	6.88	< 0.001***

*Note:* ⸱*p* < 0.1; *p < 0.05; **p < 0.01; ***p < 0.001.

The abundance of small bees and hoverflies increased significantly in the second sampling round (small bees: *E* = 0.48 ± 0.16, *p* = 0.002; hoverflies: *E* = 0.59 ± 0.27, *p* = 0.029; Table 2), and even more strongly in the third sampling round, particularly for hoverflies (small bees: *E* = 0.44 ± 0.16, *p* = 0.006; hoverflies: *E* = 1.66 ± 0.24, *p* < 0.001; Table 2). In contrast, the abundance of large bees and *Bombus* spp. did not vary among sampling rounds. Pollinator abundance also varied in relation to landscape variables. The abundance of *Bombus* spp. decreased significantly with increasing surface of artificial areas (*E* = −0.40 ± 0.17, *p* = 0.021; Table 2), while the abundance of small and large wild bees decreased significantly with increasing surface of non‐flowering crops (small bees: *E* = −0.22 ± 0.08, *p* = 0.004; large bees: *E* = −0.30 ± 0.11, *p* = 0.007; Table 2). In contrast, small bee abundance increased significantly with the surface of flowering crops (*E* = 0.18 ± 0.08, *p* = 0.023; Table 2).

Overall, a substantial proportion of the variation in abundance remained unexplained by the model with the lowest AIC for each morphogroup. The environmental and temporal variables retained in these models explained more variation for small bees (*R* ^2^ *m* = 0.23, *R* ^2^ *c* = 0.24; Table 2) and hoverflies (*R* ^2^ *m* = 0.20, *R* ^2^ *c* = 0.35) than for *Bombus* spp. (*R* ^2^ *m* = 0.03, *R* ^2^ *c* = 0.05) and large bees (*R* ^2^ *m* = 0.04, *R* ^2^ *c* = 0.05).

### 3.4 Pollinator Abundance in Blackcurrant Bushes Is Not Influenced by Flowers but Increases With Pollinator Abundance in the Adjacent Inter‐Rows

**FIGURE 5:**
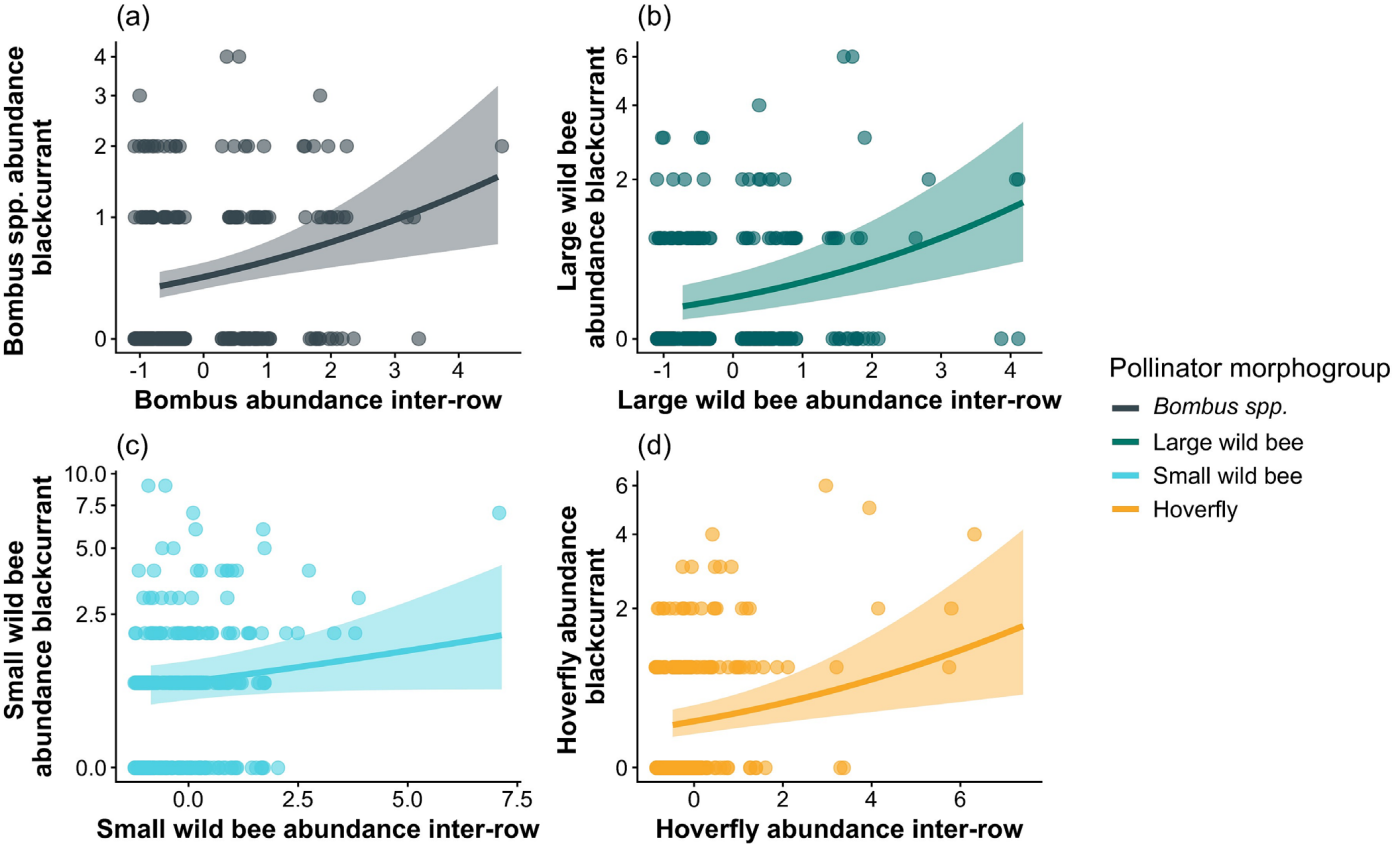
Pollinator abundance in the blackcurrant bushes (blackcurrant transect) compared to pollinator abundance in adjacent inter‐row (inter‐row transect) for (a) Bombus spp. (dark grey), (b) large wild bees (dark blue), (c) small wild bees (light blue), and (d) hoverflies (yellow). Solid lines show model predictions, while shaded areas represent 95% confidence intervals. See Table S5 for model details. Y‐axes are log_10_(*y* + 1) transformed, and explanatory variables (pollinator abundance in each morphogroup) are scaled.

Pollinator abundance in blackcurrant transects significantly increased with pollinator abundance of the same morphogroup in the adjacent inter‐row transects for *Bombus* spp. (Estimate = 0.28 ± 0.08, *p* < 0.001; Figure 5a and Table S5), large wild bees (Estimate = 0.39 ± 0.08, *p* < 0.001; Figure 5b and Table S5), and hoverflies (Estimate = 0.20 ± 0.06, *p* < 0.001; Figure 5d and Table S5). For small wild bees, we only detected a positive but non‐significant trend (Estimate = 0.10 ± 0.05, *p* = 0.065; Figure 5c and Table S5). The abundance of sown and spontaneous flowers in the inter‐rows was never retained after model selection and was not significant when added to the models with the lowest AIC (all *p* > 0.05).

## 4 Discussion

Sowing flower strips and preserving spontaneous flowers are often recommended practices to support pollinators in fragmented agricultural landscapes. However, the effectiveness of these measures and their influence on pollinator activity within orchards remain poorly understood, particularly for blackcurrant, an insect‐dependent crop. First, we showed that floral communities shifted rapidly over the three‐week study period, with taxa from the seed mixture flowering only in the inter‐rows sown in spring 2022. Moreover, our findings indicated that pollinator morphogroups in inter‐row transects responded differently to the number of spontaneous and sown flowers, as well as to transect position within inter‐rows (edge, inner, centre). The abundance of small and large wild bees increased with the number of spontaneous flowers, whereas hoverfly abundance increased with the number of sown flowers. Small wild bees, and to a lesser extent large wild bees, were less abundant in the inner and central inter‐row transects than at the edges. Finally, pollinator abundance in blackcurrant transects was not influenced by the abundance of nearby flowers, whether sown or spontaneous, but increased with pollinator abundance in adjacent inter‐row transects. This pattern was consistent for *Bombus* spp., large wild bees, and hoverflies, while the response of small wild bees only showed a positive but non‐significant trend.

### 4.1 Contrasting Benefits of Sown and Spontaneous Flowers Between Pollinator Morphogroups

Our results indicated that pollinator morphogroups in the inter‐rows responded differently to sown and spontaneous flower abundance in blackcurrant orchards. The abundance of small and large wild bees increased with the number of spontaneous flowers, whereas the abundance of hoverflies increased with the number of sown flowers. Several other publications have highlighted the benefits of sowing flower strips for hoverflies (Kohler et al. 2008; Haenke et al. 2009; Jönsson et al. 2015; Tschumi et al. 2016; von Königslöw et al. 2022; Hussain et al. 2023), even though the seed mixture compositions differed between studies. Here, hoverfly abundance increased markedly across sampling rounds (from *n* = 21 and *n* = 40 to *n* = 149 in round 3), coinciding with the progressive flowering of several sown species, particularly *Borago officinalis, Leucanthemum vulgare, Sanguisorba minor*, and *Trifolium incarnatum* . These plants likely provided suitable floral resources for hoverflies, as the flowers of *L. vulgare* , *B. officinalis* , and *S. minor* offer easily accessible pollen and nectar (Van Rijn and Wäckers 2016; Abrahamczyk et al. 2023), which can be efficiently exploited by hoverfly species which are typically short‐tongued (Cook et al. 2020). In addition, Cole et al. (2022) demonstrated increased hoverfly abundance in small‐plot fields planted exclusively with *T. incarnatum* or mixed *Trifolium* species compared to other legume crops, highlighting its potential as a floral resource for this taxon.

In contrast, small and large wild bee abundances were positively associated with the number of spontaneous flowers. This suggests that allowing local wildflowers to develop naturally within orchards may provide greater benefits for wild bees than the sown flowers from our seed mixture during early‐mid spring. Yet, the benefit of sown flower strips for bees had been demonstrated in several previous studies (Scheper et al. 2015; Buhk et al. 2018; von Königslöw et al. 2022; Schmied et al. 2022). However, the composition of the seed mixtures used in such studies varies depending on their design and objectives. In our case, it was specifically developed to provide floral resources until September and may therefore contribute more substantially to supporting wild bees later in the season, especially after blackcurrant flowering. In this regard, von Königslöw et al. (2022) found that the benefit of sown flower strips to solitary bees increased over the season and peaked in June–July, coinciding with maximum flower abundance in the strip during summer as reported in other similar studies (Ouvrard et al. 2018; Neumüller et al. 2021).

Overall, these results suggest that the complementarity of floral resources may benefit different pollinator morphogroups in blackcurrant orchards in early‐mid spring. In this study, the flower strip sown in spring 2022 resulted in a distinct flower community and greatly increased the local quantity of floral resources compared with unsown conditions (16,087 of the 16,116 floral units from species included in the seed mixture were recorded only in sown inter‐rows). However, seed mixtures are unlikely to encompass the entire diversity of flowering plants required to support pollinators with different resource needs and preferences. It is therefore necessary to also preserve spontaneous floral resources provided by species not included in the seed mixtures to fully support pollinator communities, particularly specialist species. As shown by von Königslöw et al. (2022), semi‐natural habitats are necessary for several solitary and oligolectic bee species, whose host plants are present there but absent from the flower strips.

### 4.2 Lower Wild Bee Abundance in Inner and Centre Inter‐Row Transects

Our findings revealed that the abundance of small and large wild bees decreased from edge to inner inter‐row transects, with an even stronger decline in the centre, which is in line with several previous studies across various crops (Bailey et al. 2014; Widhiono and Sudiana 2016; Ganser et al. 2018; MacInnis et al. 2020). The presence of semi‐natural habitats around orchards providing nesting and mating sites, as well as abundant spontaneous floral resources with which wild bees were positively associated in this study, may explain their higher abundance near these habitats at the edges of inter‐rows (Bailey et al. 2014; Tschanz et al. 2023). We also found that the decline in wild bee abundance toward the inner and central inter‐row transects was more pronounced for small bees than for larger ones, suggesting more limited spillover of this morphogroup within the inter‐rows. MacInnis et al. (2020) similarly reported a significant decline in small wild bee abundance in strawberry field centres, which may be explained by their limited foraging range (Greenleaf et al. 2007), restricting their ability to disperse from inter‐row margins toward the interior. Subdividing large blackcurrant orchards or incorporating linear habitats such as woody hedgerows (Kremen et al. 2019; Mignot 2023; Bishop et al. 2023) or well‐established wildflower strips that meet wild bee requirements (von Königslöw et al. 2022; Schmied et al. 2022) could provide ecological corridors and facilitate access to the whole orchard. This could also enhance overall orchard pollination, as Ganser et al. (2018) reported higher pollination services at the edges of strawberry crops than in the centre, although this pattern is not systematic (Woodcock et al. 2016; Albrecht et al. 2020).

In contrast, the abundance of *Bombus* spp. and hoverflies did not vary between edge, inner, and central inter‐row transects. For bumblebees, these results are consistent with Sõber et al. (2025) and may be explained by their ability to forage over long distances across diverse floral resources, as well as by their relatively flexible nesting site requirements compared with other pollinators (Westphal et al. 2003; Redhead et al. 2016). Blackcurrant flowers likely provided abundant nectar and pollen during flowering and may have attracted bumblebees from surrounding semi‐natural habitats throughout the orchard, regardless of distance from the edge. Conversely, hoverflies are not central‐place foragers like bees, which must collect pollen and nectar to feed their offspring and frequently return to their nests (Jauker et al. 2009). As a result, hoverflies can easily disperse over long distances, even among smaller species (Doyle et al. 2020). Similar results have been reported by McCravy et al. (2024) in apple and peach orchards, with no difference in hoverfly abundance and species richness between orchard edges and interiors.

### 4.3 Pollinator Abundance in Blackcurrant Bushes Is Not Influenced by Flowers but Increases With Pollinator Abundance in the Adjacent Inter‐Rows

Our results indicated that for *Bombus* spp., large wild bees, and hoverflies, pollinator abundance in blackcurrant bushes significantly increased with the abundance of the same pollinator morphogroup in the adjacent inter‐rows. For small wild bees, we only detected a slight positive trend. Overall, these findings suggest that implementing suitable agroecological measures to increase pollinator abundance in inter‐rows could also enhance pollinator abundance in blackcurrant bushes. In addition, we did not detect a significant effect of the abundance of sown or spontaneous flowers in the inter‐rows on the abundance of any pollinator morphogroup in the blackcurrant bushes. Although this may suggest that nearby floral resources did not influence pollinator activity in blackcurrant bushes, our approach does not allow us to determine whether direct competition or facilitation for pollinators occurs between blackcurrant flowers and adjacent floral resources. Future research should therefore focus on pollinators actively visiting flowers (sown, spontaneous, and blackcurrant) and measure blackcurrant fruit traits (e.g., fruit set, seed number, fruit mass, and fruit quality) (Bartholomée and Lavorel 2019), to provide more direct insights into potential competition for pollinators and the benefits of these conservation measures for blackcurrant pollination. In their study in almond orchards, Klein et al. (2012) reported higher visitation frequencies of wild bees and hoverflies on almond flowers in orchards adjacent to riparian vegetation strips and in those located in landscapes containing a greater proportion of semi‐natural habitats. This highlights the importance of providing suitable habitats that offer not only floral resources but also non‐floral elements such as nesting and protective materials to fully support pollinator communities and enhance pollination services in crops (Bailey et al. 2014; Requier and Leonhardt 2020; Ganser et al. 2021).

## Author Contributions

**Mathieu Lachaise:** conceptualization (equal), data curation (lead), formal analysis (lead), investigation (equal), methodology (equal), project administration (supporting), resources (equal), software (lead), supervision (equal), visualization (lead), writing – original draft (lead), writing – review and editing (equal). **Blanche Collard:** conceptualization (equal), data curation (supporting), formal analysis (supporting), software (supporting), supervision (equal), visualization (supporting), writing – original draft (supporting), writing – review and editing (equal). **Marie‐Charlotte Anstett:** conceptualization (equal), data curation (supporting), formal analysis (supporting), funding acquisition (lead), investigation (equal), methodology (equal), project administration (lead), resources (equal), software (supporting), supervision (equal), visualization (supporting), writing – original draft (supporting), writing – review and editing (equal).

## Funding

This research was supported by EIP‐AGRI (EU CAP Network), FEADER, and the Bourgogne Franche‐Comté region through the following grants awarded to MCA: FEADER RBOU160118CR0260011 2018–2021 and FEADER RBOU160121CR0260003 2022–2023, as part of the project *Pérennité et développement de la filière cassis en Bourgogne*.

## Ethics Statement

The authors have nothing to report.

## Consent

The authors have nothing to report.

## Conflicts of Interest

The authors declare no conflicts of interest.

## Supporting information


**Figure S1:** Maps of the landscape composition within a 500‐m radius for the nine sites.
**Figure S2:** Correlation plot of environmental variables tested for model selection.
**Table S1:** Composition of the seed mix used for the flower strips.
**Table S2:** List of sown and spontaneous flowering plants identified in blackcurrant orchards.
**Table S3:** Description of each landscape category.
**Table S4:** List of pollinators identified in blackcurrant orchards.
**Table S5:** GLMMs of the effects of pollinator and flower abundance in inter‐row spaces (inter‐row transects) with pollinator abundance in blackcurrant bushes (blackcurrant transects) for each morphogroup.

## Data Availability

The dataset used in this study is openly available on Mendeley Data: https://doi.org/10.17632/8jtpwp7ntf.1. The scripts used for data analysis can be made available upon request from the corresponding author (M. Lachaise, email: mathieu.lachaise.pro@gmail.com).

## References

[ece374127-bib-0001] Abrahamczyk, S. , J. Struck , and M. Weigend . 2023. “Pollination Mode and Reproductive System of * sanguisorba minor* and * s * *anguisorba officinalis* .” Plant Species Biology 38: 171–179. 10.1111/1442-1984.12404.

[ece374127-bib-0002] Albrecht, M. , D. Kleijn , N. M. Williams , et al. 2020. “The Effectiveness of Flower Strips and Hedgerows on Pest Control, Pollination Services and Crop Yield: A Quantitative Synthesis.” Ecology Letters 23: 1488–1498. 10.1111/ele.13576. 32808477 PMC7540530

[ece374127-bib-0003] Albrecht, M. , A. Knecht , M. Riesen , T. Rutz , and D. Ganser . 2021. “Time Since Establishment Drives Bee and Hoverfly Diversity, Abundance of Crop‐Pollinating Bees and Aphidophagous Hoverflies in Perennial Wildflower Strips.” Basic and Applied Ecology 57: 102–114. 10.1016/j.baae.2021.10.003.

[ece374127-bib-0004] Ammann, L. , A. Bosem‐Baillod , F. Herzog , D. Frey , M. H. Entling , and M. Albrecht . 2024. “Spatio‐Temporal Complementarity of Floral Resources Sustains Wild Bee Pollinators in Agricultural Landscapes.” Agriculture, Ecosystems & Environment 359: 108754. 10.1016/j.agee.2023.108754.

[ece374127-bib-0005] Anstett, M. C. , M. Duchet‐Annez , and C. Denis . 2022. Projet Cassis: Approche Pluridisciplinaire Pour des Mesures Agro Écologiques. Bourgogne‐Franche‐Comté Nature.

[ece374127-bib-0006] Anstett, M. C. , M. Léon de Treveret , and P. Louapre . 2019. “La Pollinisation du Cassis: État des Lieux Dans un Contexte de Changements Anthropiques.” Bourgogne‐Franche‐Comté Nature 29: 194–205.

[ece374127-bib-0007] Azpiazu, C. , P. Medina , Á. Adán , et al. 2020. “The Role of Annual Flowering Plant Strips on a Melon Crop in Central Spain. Influence on Pollinators and Crop.” Insects 11: 66. 10.3390/insects11010066. 31968621 PMC7022770

[ece374127-bib-0008] Bailey, S. , F. Requier , B. Nusillard , S. P. M. Roberts , S. G. Potts , and C. Bouget . 2014. “Distance From Forest Edge Affects Bee Pollinators in Oilseed Rape Fields.” Ecology and Evolution 4: 370–380. 10.1002/ece3.924. 24634722 PMC3936384

[ece374127-bib-0009] Baldock, K. C. R. , M. A. Goddard , D. M. Hicks , et al. 2015. “Where Is the UK's Pollinator Biodiversity? The Importance of Urban Areas for Flower‐Visiting Insects.” Proceedings of the Royal Society B: Biological Sciences 282: 20142849. 10.1098/rspb.2014.2849. PMC4345454 25673686

[ece374127-bib-0010] Bartholomée, O. , and S. Lavorel . 2019. “Disentangling the Diversity of Definitions for the Pollination Ecosystem Service and Associated Estimation Methods.” Ecological Indicators 107: 105576. 10.1016/j.ecolind.2019.105576.

[ece374127-bib-0011] Bartoń, K. 2024. “MuMIn: Multi‐Model Inference.” R Package Version 1.48.4. https://CRAN.R‐project.org/package=MuMIn.

[ece374127-bib-0012] Bascompte, J. , and M. Scheffer . 2023. “The Resilience of Plant–Pollinator Networks.” Annual Review of Entomology 68: 363–380. 10.1146/annurev-ento-120120-102424. 36206771

[ece374127-bib-0013] Bishop, G. A. , T. P. M. Fijen , B. N. Desposato , J. Scheper , and D. Kleijn . 2023. “Hedgerows Have Contrasting Effects on Pollinators and Natural Enemies and Limited Spillover Effects on Apple Production.” Agriculture, Ecosystems & Environment 346: 108364. 10.1016/j.agee.2023.108364.

[ece374127-bib-0014] Bottero, I. , C. Dominik , O. Schweiger , et al. 2023. “Impact of Landscape Configuration and Composition on Pollinator Communities Across Different European Biogeographic Regions.” Frontiers in Ecology and Evolution 11: 1128228. 10.3389/fevo.2023.1128228.

[ece374127-bib-0015] Brooks, M. E. , K. Kristensen , K. J. van Benthem , et al. 2017. “glmmTMB Balances Speed and Flexibility Among Packages for Zero‐Inflated Generalized Linear Mixed Modeling.” R Journal 9, no. 2: 378–400. 10.32614/RJ-2017-066.

[ece374127-bib-0016] Buhk, C. , R. Oppermann , A. Schanowski , R. Bleil , J. Lüdemann , and C. Maus . 2018. “Flower Strip Networks Offer Promising Long Term Effects on Pollinator Species Richness in Intensively Cultivated Agricultural Areas.” BMC Ecology 18: 55. 10.1186/s12898-018-0210-z. 30514253 PMC6280486

[ece374127-bib-0017] Campbell, A. J. , A. Wilby , P. Sutton , and F. L. Wäckers . 2017. “Do Sown Flower Strips Boost Wild Pollinator Abundance and Pollination Services in a Spring‐Flowering Crop? A Case Study From UK Cider Apple Orchards.” Agriculture, Ecosystems & Environment 239: 20–29. 10.1016/j.agee.2017.01.005.

[ece374127-bib-0018] Carturan, B. S. , N. Siewe , C. A. Cobbold , and R. C. Tyson . 2023. “Bumble Bee Pollination and the Wildflower/Crop Trade‐Off: When Do Wildflower Enhancements Improve Crop Yield?” Ecological Modelling 484: 110447. 10.1016/j.ecolmodel.2023.110447.

[ece374127-bib-0019] Cole, L. J. , J. A. Baddeley , D. Robertson , C. F. E. Topp , R. L. Walker , and C. A. Watson . 2022. “Supporting Wild Pollinators in Agricultural Landscapes Through Targeted Legume Mixtures.” Agriculture, Ecosystems & Environment 323: 107648. 10.1016/j.agee.2021.107648. PMC8591731 34980933

[ece374127-bib-0020] Cook, D. F. , S. C. Voss , J. T. D. Finch , R. C. Rader , J. M. Cook , and C. J. Spurr . 2020. “The Role of Flies as Pollinators of Horticultural Crops: An Australian Case Study With Worldwide Relevance.” Insects 11: 341. 10.3390/insects11060341. 32498457 PMC7349676

[ece374127-bib-0021] Desaegher, J. , D. Sheeren , and A. Ouin . 2021. “Optimising Spatial Distribution of Mass‐Flowering Patches at the Landscape Scale to Increase Crop Pollination.” Journal of Applied Ecology 58: 1876–1887. 10.1111/1365-2664.13949.

[ece374127-bib-0022] Doyle, T. , W. L. S. Hawkes , R. Massy , G. D. Powney , M. H. M. Menz , and K. R. Wotton . 2020. “Pollination by Hoverflies in the Anthropocene.” Proceedings of the Royal Society B: Biological Sciences 287: 20200508. 10.1098/rspb.2020.0508. PMC7287354 32429807

[ece374127-bib-0023] Eeraerts, M. , S. Van Den Berge , W. Proesmans , et al. 2021. “Fruit Orchards and Woody Semi‐Natural Habitat Provide Complementary Resources for Pollinators in Agricultural Landscapes.” Landscape Ecology 36: 1377–1390. 10.1007/s10980-021-01220-y.

[ece374127-bib-0024] Encinas‐Viso, F. , J. Bovill , D. E. Albrecht , et al. 2023. “Pollen DNA Metabarcoding Reveals Cryptic Diversity and High Spatial Turnover in Alpine Plant–Pollinator Networks.” Molecular Ecology 32: 6377–6393. 10.1111/mec.16682. 36065738

[ece374127-bib-0025] FAO . 2024. “Pesticides Use and Trade—1990–2022.” FAOSTAT Analytical Briefs, No. 89. Rome.

[ece374127-bib-0026] Feltham, H. , K. Park , J. Minderman , and D. Goulson . 2015. “Experimental Evidence That Wildflower Strips Increase Pollinator Visits to Crops.” Ecology and Evolution 5: 3523–3530. 10.1002/ece3.1444. 26380683 PMC4569045

[ece374127-bib-0027] Ganser, D. , M. Albrecht , and E. Knop . 2021. “Wildflower Strips Enhance Wild Bee Reproductive Success.” Journal of Applied Ecology 58: 486–495. 10.1111/1365-2664.13778.

[ece374127-bib-0028] Ganser, D. , B. Mayr , M. Albrecht , and E. Knop . 2018. “Wildflower Strips Enhance Pollination in Adjacent Strawberry Crops at the Small Scale.” Ecology and Evolution 8: 11775–11784. 10.1002/ece3.4631. 30598775 PMC6303775

[ece374127-bib-0029] Goulson, D. , E. Nicholls , C. Botías , and E. L. Rotheray . 2015. “Bee Declines Driven by Combined Stress From Parasites, Pesticides, and Lack of Flowers.” Science 347: 1255957. 10.1126/science.1255957. 25721506

[ece374127-bib-0030] Greenleaf, S. S. , N. M. Williams , R. Winfree , and C. Kremen . 2007. “Bee Foraging Ranges and Their Relationship to Body Size.” Oecologia 153: 589–596. 10.1007/s00442-007-0752-9. 17483965

[ece374127-bib-0031] Haenke, S. , B. Scheid , M. Schaefer , T. Tscharntke , and C. Thies . 2009. “Increasing Syrphid Fly Diversity and Density in Sown Flower Strips Within Simple vs. Complex Landscapes.” Journal of Applied Ecology 46: 1106–1114. 10.1111/j.1365-2664.2009.01685.x.

[ece374127-bib-0032] Hartig, F. 2022. “DHARMa: Residual Diagnostics for Hierarchical (Multi‐Level/Mixed) Regression Models.” R Package Version 0.4.6. https://CRAN.R‐project.org/package=DHARMa.

[ece374127-bib-0033] Hussain, R. I. , R. Walcher , N. Vogel , B. Krautzer , L. Rasran , and T. Frank . 2023. “Effectiveness of Flowers Strips on Insect's Restoration in Intensive Grassland.” Agriculture, Ecosystems & Environment 348: 108436. 10.1016/j.agee.2023.108436.

[ece374127-bib-0034] Jauker, F. , T. Diekötter , F. Schwarzbach , and V. Wolters . 2009. “Pollinator Dispersal in an Agricultural Matrix: Opposing Responses of Wild Bees and Hoverflies to Landscape Structure and Distance From Main Habitat.” Landscape Ecology 24: 547–555. 10.1007/s10980-009-9331-2.

[ece374127-bib-0035] Jönsson, A. M. , J. Ekroos , J. Dänhardt , G. K. S. Andersson , O. Olsson , and H. G. Smith . 2015. “Sown Flower Strips in Southern Sweden Increase Abundances of Wild Bees and Hoverflies in the Wider Landscape.” Biological Conservation 184: 51–58. 10.1016/j.biocon.2014.12.027.

[ece374127-bib-0036] Junqueira, C. N. , R. A. S. Pereira , R. C. da Silva , et al. 2022. “Do Apis and Non‐ Bees Provide a Similar Contribution to Crop Production With Different Levels of Pollination Dependency? A Review Using Meta‐Analysis.” Ecological Entomology 47: 76–83. 10.1111/een.13092.

[ece374127-bib-0037] Katumo, D. M. , H. Liang , A. C. Ochola , M. Lv , Q. F. Wang , and C. F. Yang . 2022. “Pollinator Diversity Benefits Natural and Agricultural Ecosystems, Environmental Health, and Human Welfare.” Plant Diversity 44: 429–435. 10.1016/j.pld.2022.01.005. 36187551 PMC9512639

[ece374127-bib-0038] Kennedy, C. M. , E. Lonsdorf , M. C. Neel , et al. 2013. “A Global Quantitative Synthesis of Local and Landscape Effects on Wild Bee Pollinators in Agroecosystems.” Ecology Letters 16: 584–599. 10.1111/ele.12082. 23489285

[ece374127-bib-0039] Kleijn, D. , R. Winfree , I. Bartomeus , et al. 2015. “Delivery of Crop Pollination Services Is an Insufficient Argument for Wild Pollinator Conservation.” Nature Communications 6: 7414. 10.1038/ncomms8414. PMC4490361 26079893

[ece374127-bib-0041] Klein, A. M. , B. E. Vaissière , J. H. Cane , et al. 2007. “Importance of Pollinators in Changing Landscapes for World Crops.” Proceedings of the Royal Society B 274: 303–313. 10.1098/rspb.2006.3721. 17164193 PMC1702377

[ece374127-bib-0040] Klein, A.‐M. , C. Brittain , S. D. Hendrix , R. Thorp , N. Williams , and C. Kremen . 2012. “Wild Pollination Services to California Almond Rely on Semi‐Natural Habitat.” Journal of Applied Ecology 49: 723–732. 10.1111/j.1365-2664.2012.02144.x.

[ece374127-bib-0042] Knapp, J. L. , R. F. Shaw , and J. L. Osborne . 2019. “Pollinator Visitation to Mass‐Flowering Courgette and Co‐Flowering Wild Flowers: Implications for Pollination and Bee Conservation on Farms.” Basic and Applied Ecology 34: 85–94. 10.1016/j.baae.2018.09.003.

[ece374127-bib-0043] Kohler, F. , J. Verhulst , R. Van Klink , and D. Kleijn . 2008. “At What Spatial Scale Do High‐Quality Habitats Enhance the Diversity of Forbs and Pollinators in Intensively Farmed Landscapes?” Journal of Applied Ecology 45: 753–762. 10.1111/j.1365-2664.2007.01394.x.

[ece374127-bib-0044] Kremen, C. , M. Albrecht , and L. Ponisio . 2019. “Restoring Pollinator Communities and Pollination Services in Hedgerows in Intensively Managed Agricultural Landscapes.” In The Ecology of Hedgerows and Field Margins. Routledge.

[ece374127-bib-0045] Krimmer, E. , E. A. Martin , J. Krauss , A. Holzschuh , and I. Steffan‐Dewenter . 2019. “Size, Age and Surrounding Semi‐Natural Habitats Modulate the Effectiveness of Flower‐Rich Agri‐Environment Schemes to Promote Pollinator Visitation in Crop Fields.” Agriculture, Ecosystems & Environment 284: 106590. 10.1016/j.agee.2019.106590.

[ece374127-bib-0046] Le Féon, V. , F. Burel , R. Chifflet , et al. 2013. “Solitary Bee Abundance and Species Richness in Dynamic Agricultural Landscapes.” Agriculture, Ecosystems & Environment 166: 94–101. 10.1016/j.agee.2011.06.020.

[ece374127-bib-0047] Lowe, E. B. , R. Groves , and C. Gratton . 2021. “Impacts of Field‐Edge Flower Plantings on Pollinator Conservation and Ecosystem Service Delivery—A Meta‐Analysis.” Agriculture, Ecosystems & Environment 310: 107290. 10.1016/j.agee.2020.107290.

[ece374127-bib-0048] Lüdecke, D. 2018. “Ggeffects: Tidy Data Frames of Marginal Effects From Regression Models.” Journal of Open Source Software 3, no. 26: 772. 10.21105/joss.00772.

[ece374127-bib-0049] Lüdecke, D. , M. S. Ben‐Shachar , and I. Patil . 2021. “Performance: An R Package for Assessment, Comparison and Testing of Statistical Models.” Journal of Open Source Software 6, no. 60: 3139. 10.21105/joss.03139.

[ece374127-bib-0050] MacInnis, G. , C. M. Buddle , and J. R. K. Forrest . 2020. “Small Wild Bee Abundance Declines With Distance Into Strawberry Crops Regardless of Field Margin Habitat.” Basic and Applied Ecology 44: 14–23. 10.1016/j.baae.2020.02.007.

[ece374127-bib-0051] Maurer, C. , L. Sutter , C. Martínez‐Núñez , L. Pellissier , and M. Albrecht . 2022. “Different Types of Semi‐Natural Habitat Are Required to Sustain Diverse Wild Bee Communities Across Agricultural Landscapes.” Journal of Applied Ecology 59: 2604–2615. 10.1111/1365-2664.14260.

[ece374127-bib-0052] McCravy, K. W. , C. S. Clem , J. B. Bailey , et al. 2024. “Hover Fly (Diptera: Syrphidae) Diversity and Seasonality in North Georgia Apple and Peach Orchards.” Journal of Economic Entomology 117: 1572–1581. 10.1093/jee/toae103. 38779977 PMC11318625

[ece374127-bib-0053] Mignot, M. 2023. “BILAN—Inventaires des Abeilles Sauvages Dans les Cassissiers de Bourgogne Franche‐Comté.”

[ece374127-bib-0083] Nakagawa, S. , P. C. D. Johnson , and H. Schielzeth . 2017. “The Coefficient of Determination R 2 and Intra‐Class Correlation Coefficient from Generalized Linear Mixed‐Effects Models Revisited and Expanded.” Journal of the Royal Society Interface 14, no. 134: 20170213. 10.1098/rsif.2017.0213. 28904005 PMC5636267

[ece374127-bib-0054] Neira, P. , J. M. Blanco‐Moreno , M. Olave , B. Caballero‐López , and F. X. Sans . 2024. “Effects of Agricultural Landscape Heterogeneity on Pollinator Visitation Rates in Mediterranean Oilseed Rape.” Agriculture, Ecosystems & Environment 363: 108869. 10.1016/j.agee.2023.108869.

[ece374127-bib-0055] Neumüller, U. , H. Burger , H. R. Schwenninger , et al. 2021. “Prolonged Blooming Season of Flower Plantings Increases Wild Bee Abundance and Richness in Agricultural Landscapes.” Biodiversity and Conservation 30: 3003–3021. 10.1007/s10531-021-02233-4.

[ece374127-bib-0056] Oksanen, J. , G. Simpson , F. Blanchet , et al. 2022. “Vegan: Community Ecology Package.” R Package Version 2.6‐4. https://CRAN.R‐project.org/package=vegan.

[ece374127-bib-0057] Ollerton, J. , R. Winfree , and S. Tarrant . 2011. “How Many Flowering Plants Are Pollinated by Animals?” Oikos 120: 321–326. 10.1111/j.1600-0706.2010.18644.x.

[ece374127-bib-0058] Ouvrard, P. , J. Transon , and A.‐L. Jacquemart . 2018. “Flower‐Strip Agri‐Environment Schemes Provide Diverse and Valuable Summer Flower Resources for Pollinating Insects.” Biodiversity and Conservation 27: 2193–2216. 10.1007/s10531-018-1531-0.

[ece374127-bib-0059] Potts, S. G. , J. C. Biesmeijer , C. Kremen , P. Neumann , O. Schweiger , and W. E. Kunin . 2010. “Global Pollinator Declines: Trends, Impacts and Drivers.” Trends in Ecology & Evolution 25: 345–353. 10.1016/j.tree.2010.01.007. 20188434

[ece374127-bib-0060] Potts, S. G. , V. L. Imperatriz‐Fonseca , H. T. Ngo , et al. 2016. The Assessment Report on Pollinators, Pollination and Food Production: Summary for Policymakers. Secretariat of the Intergovernmental Science‐Policy Platform on Biodiversity and Ecosystem Services.

[ece374127-bib-0085] R Core Team . 2023. R: A Language and Environment for Statistical Computing Version 4.3.2. R Foundation for Statistical Computing.

[ece374127-bib-0061] Redhead, J. W. , S. Dreier , A. F. G. Bourke , et al. 2016. “Effects of Habitat Composition and Landscape Structure on Worker Foraging Distances of Five Bumble Bee Species.” Ecological Applications 26: 726–739. 10.1890/15-0546. 27411246

[ece374127-bib-0062] Requier, F. , and S. D. Leonhardt . 2020. “Beyond Flowers: Including Non‐Floral Resources in Bee Conservation Schemes.” Journal of Insect Conservation 24: 5–16. 10.1007/s10841-019-00206-1.

[ece374127-bib-0063] Rohde, A. T. , and D. S. Pilliod . 2021. “Spatiotemporal Dynamics of Insect Pollinator Communities in Sagebrush Steppe Associated With Weather and Vegetation.” Global Ecology and Conservation 29: e01691. 10.1016/j.gecco.2021.e01691.

[ece374127-bib-0064] Rousset, F. , and J. Ferdy . 2014. “Testing Environmental and Genetic Effects in the Presence of Spatial Autocorrelation.” Ecography 37, no. 8: 781–790. 10.1111/ecog.00566.

[ece374127-bib-0065] Scheper, J. , R. Bommarco , A. Holzschuh , et al. 2015. “Local and Landscape‐Level Floral Resources Explain Effects of Wildflower Strips on Wild Bees Across Four European Countries.” Journal of Applied Ecology 52: 1165–1175. 10.1111/1365-2664.12479.

[ece374127-bib-0066] Scheper, J. , T. Bukovinszky , M. E. Huigens , and D. Kleijn . 2021. “Attractiveness of Sown Wildflower Strips to Flower‐Visiting Insects Depends on Seed Mixture and Establishment Success.” Basic and Applied Ecology 56: 401–415. 10.1016/j.baae.2021.08.014.

[ece374127-bib-0067] Scheper, J. , A. Holzschuh , M. Kuussaari , et al. 2013. “Environmental Factors Driving the Effectiveness of European Agri‐Environmental Measures in Mitigating Pollinator Loss—A Meta‐Analysis.” Ecology Letters 16: 912–920. 10.1111/ele.12128. 23714393

[ece374127-bib-0068] Schmied, H. , L. Getrost , O. Diestelhorst , G. Maaßen , and L. Gerhard . 2022. “Between Perfect Habitat and Ecological Trap: Even Wildflower Strips Mulched Annually Increase Pollinating Insect Numbers in Intensively Used Agricultural Landscapes.” Journal of Insect Conservation 26: 425–434. 10.1007/s10841-022-00383-6.

[ece374127-bib-0069] Schulte, K. D. , C. J. Wilson , A. Tawril , and M. A. Jamieson . 2025. “Spatiotemporal Variability and Functional Redundancy Obscure Effects of Urbanization on Strawberry Pollinators.” Ecosphere 16: e70133. 10.1002/ecs2.70133.

[ece374127-bib-0070] Senapathi, D. , M. A. Goddard , W. E. Kunin , and K. C. R. Baldock . 2017. “Landscape Impacts on Pollinator Communities in Temperate Systems: Evidence and Knowledge Gaps.” Functional Ecology 31: 26–37. 10.1111/1365-2435.12809.

[ece374127-bib-0071] Sõber, V. , M.‐L. Viljur , V. Soon , A. Kaasik , T. Aavik , and T. Teder . 2025. “No Evidence of Decline in Bumblebee Abundance and Species Richness From Edge to Interior in Moderately‐Sized Red Clover Fields.” Landscape Ecology 40: 185. 10.1007/s10980-025-02205-x.

[ece374127-bib-0084] Steffan‐Dewenter, I. , U. Münzenberg , C. Bürger , C. Thies , and T. Tscharntke . 2002. “Scale‐Dependent Effects of Landscape Context on Three Pollinator Guilds.” Ecology 83, no. 5: 1421–1432. 10.1890/0012-9658(2002)083[1421:sdeolc]2.0.co;2.

[ece374127-bib-0072] Tschanz, P. , S. Vogel , A. Walter , T. Keller , and M. Albrecht . 2023. “Nesting of Ground‐Nesting Bees in Arable Fields Is Not Associated With Tillage System Per Se, but With Distance to Field Edge, Crop Cover, Soil and Landscape Context.” Journal of Applied Ecology 60: 158–169. 10.1111/1365-2664.14317.

[ece374127-bib-0073] Tschumi, M. , M. Albrecht , J. Collatz , et al. 2016. “Tailored Flower Strips Promote Natural Enemy Biodiversity and Pest Control in Potato Crops.” Journal of Applied Ecology 53: 1169–1176. 10.1111/1365-2664.12653.

[ece374127-bib-0074] Turo, K. J. , J. R. Reilly , T. P. M. Fijen , A. Magrach , and R. Winfree . 2024. “Insufficient Pollinator Visitation Often Limits Yield in Crop Systems Worldwide.” Nature Ecology & Evolution 8: 1612–1622. 10.1038/s41559-024-02460-2. 38961256

[ece374127-bib-0075] Van Rijn, P. C. J. , and F. L. Wäckers . 2016. “Nectar Accessibility Determines Fitness, Flower Choice and Abundance of Hoverflies That Provide Natural Pest Control.” Journal of Applied Ecology 53: 925–933. 10.1111/1365-2664.12605.

[ece374127-bib-0076] Vanbergen, A. J. , M. Baude , and J. C. Biesmeijer . 2013. “Threats to an Ecosystem Service: Pressures on Pollinators.” Frontiers in Ecology and the Environment 11: 251–259. 10.1890/120126.

[ece374127-bib-0077] von Königslöw, V. , F. Fornoff , and A.‐M. Klein . 2022. “Pollinator Enhancement in Agriculture: Comparing Sown Flower Strips, Hedges and Sown Hedge Herb Layers in Apple Orchards.” Biodiversity and Conservation 31: 433–451. 10.1007/s10531-021-02338-w.

[ece374127-bib-0078] Westphal, C. , I. Steffan‐Dewenter , and T. Tscharntke . 2003. “Mass Flowering Crops Enhance Pollinator Densities at a Landscape Scale.” Ecology Letters 6: 961–965. 10.1046/j.1461-0248.2003.00523.x.

[ece374127-bib-0079] Wickham, H. 2016. ggplot2: Elegant Graphics for Data Analysis. Springer‐Verlag.

[ece374127-bib-0080] Widhiono, I. , and E. Sudiana . 2016. “Impact of Distance From the Forest Edge on the Wild Bee Diversity on the Northern Slope of Mount Slamet.” Biosaintifika: Journal of Biology & Biology Education 8, no. 2: 148–154.

[ece374127-bib-0081] Woodcock, B. A. , J. M. Bullock , M. McCracken , et al. 2016. “Spill‐Over of Pest Control and Pollination Services Into Arable Crops.” Agriculture, Ecosystems & Environment 231: 15–23. 10.1016/j.agee.2016.06.023.

[ece374127-bib-0082] Zuur, A. F. , E. N. Ieno , and C. S. Elphick . 2010. “A Protocol for Data Exploration to Avoid Common Statistical Problems.” Methods in Ecology and Evolution 1, no. 1: 3–14. 10.1111/j.2041-210x.2009.00001.x.

