# Supplementary material for: Pollinators in Blackcurrant Orchards: Complementary Roles of Sown and Spontaneous Flower Strips and Limited Spillover of Small Wild Bees Within Fields
Source: Ecol Evol. 2026 Sep 10;16(9):e74127. doi: 10.1002/ece3.74127 (PMC13559713; doi:10.1002/ece3.74127)
Supplement: Supplementary file 1 [file ECE3-16-e74127-s001.docx]

**Article title:** Pollinators in blackcurrant orchards: Complementary roles of sown and spontaneous flower strips and limited spillover of small wild bees within fields

**Journal name:** Ecology and Evolution

**Mathieu Lachaise^a^*, Blanche Collard^a,b^, Marie-Charlotte Anstett^c^**

^a^ Université Paris-Saclay, CNRS, AgroParisTech, UMR Ecologie Société Evolution, 91190, Gif-sur-Yvette, France

^b^ AgroParisTech, 22 place de l'Agronomie, CS 20040, 91123 Palaiseau Cedex, France

^c^ UMR 6282 Biogéosciences, CNRS Université Bourgogne Europe 6 bd Gabriel-21 000 Dijon, France

*	corresponding author (Email: mathieu.lachaise.pro@gmail.com, ORCID: [0009-0004-7774-5219](javascript:popup_orcidDetail('https://orcid.org'%20,'0009-0004-7774-5219');))

**TABLE OF CONTENTS**

**1. Supplementary Figures**

**Fig. S1:** Maps of the landscape composition within a 500-meter radius for the nine sites

**Fig. S2:** Correlation plot of environmental variables tested for model selection.

**2. Supplementary Tables**

**Table S1:** Composition of the seed mix used for the flower strips

**Table S2:** List of sown and spontaneous flowering plants identified in blackcurrant orchards

**Table S3:** Description of each landscape category

**Table S4:** List of pollinators identified in blackcurrant orchards

**Table S5:** GLMMs of the effects of pollinator and flower abundance in inter-row spaces (inter-row transects) with pollinator abundance in blackcurrant bushes (blackcurrant transects) for each morphogroup

**1. Supplementary Figures**

**Fig. S1:** Detailed maps of the landscape composition within a 500 m radius for the nine sites. White dots correspond to the central point of our experimental design in blackcurrant orchards. For the analysis presented in this study, several landscape elements were pre-grouped, as detailed in the Materials and Methods section, in order to limit the number of landscape variables

**Fig. S2:** Correlation plot of environmental variables tested for model selection. Maximum Pearson coefficients were |r| < 0.51, indicating limited correlation among variables. Multicollinearity was also tested in the final models.

**1. Supplementary Tables**

**Table S1:** Composition of the seed mix used for the flower strips, composed of 20% local wildflower species and 80% grass species (*Poaceae)*.

| **Management** | **Species** |
| --- | --- |
| Wildflowers (20%) | *Achillea millefolium L.* |
|  | *Anthemis tinctoria L.* |
|  | *Anthyllis vulneraria L.* |
|  | *Borago officinalis L.* |
|  | *Centaurea jacea L.* |
|  | *Cyanus segetum H.* |
|  | *Echium vulgare L.* |
|  | *Fagopyrum esculentum Moench* |
|  | *Leucanthemum vulgare Lam.* |
|  | *Lotus corniculatus L.* |
|  | *Onobrychis viciifolia Scop.* |
|  | *Sanguisorba minor Scop.* |
|  | *Silene vulgaris (Moench) Garcke* |
|  | *Trifolium incarnatum L.* |
| Grasses (80%) | *Bromus erectus Huds.* |
|  | *Festuca ovina L.* |
|  | *Festuca rubra L.* |

**Table S2:** List of sown and spontaneous flowering plants identified to the genus or species level and observed in all transects within blackcurrant orchards at each sampling round. Floral abundance corresponds to the total number of inflorescences of each species or genus counted throughout our surveys.

| **Management** | **Genus or species** | **Floral abundance S1** | **Floral abundance S2** | **Floral abundance S3** | **Floral abundance**  **all rounds** |
| --- | --- | --- | --- | --- | --- |
| **Sown flowers** | *Anthemis tinctoria L.* | 0 | 0 | 2 | 2 |
|  | *Anthyllis vulneraria L.* | 0 | 0 | 30 | 30 |
|  | *Borago officinalis L.* | 191 | 325 | 532 | 1 048 |
|  | *Cyanus segetum* Hill | 0 | 0 | 2 | 2 |
|  | *Leucanthemum vulgare* Lam. | 2 | 5 | 406 | 413 |
|  | *Lotus corniculatus L.* | 0 | 1 | 81 | 82 |
|  | *Onobrychis viciifolia* Scop. | 0 | 0 | 1 | 1 |
|  | *Sanguisorba minor* Scop. | 112 | 3 246 | 10 770 | 14 128 |
|  | *Trifolium incarnatum* L. | 0 | 68 | 332 | 400 |
| **Spontaneous flowers** | *Bellis perennis* L. | 0 | 0 | 19 | 19 |
|  | *Brassica napus* L. | 1 | 4 | 40 | 45 |
|  | *Capsella* spp. | 0 | 2 | 0 | 2 |
|  | *Cardamine* spp. | 0 | 3 | 0 | 3 |
|  | *Cerastium* spp. | 253 | 22 | 81 | 356 |
|  | *Geranium* spp. | 30 | 51 | 238 | 319 |
|  | *Lamium purpureum* L. | 816 | 481 | 62 | 1 359 |
|  | *Matricaria* spp. | 0 | 153 | 406 | 559 |
|  | *Medicago* spp. | 23 | 76 | 149 | 248 |
|  | *Myosotis* spp. | 1 | 16 | 15 | 32 |
|  | *Ornithogalum* spp. | 26 | 399 | 6 | 431 |
|  | *Ranunculus* spp. | 169 | 569 | 423 | 1 161 |
|  | *Senecio* spp. | 179 | 43 | 64 | 286 |
|  | *Sonchus* spp. | 0 | 1 | 36 | 37 |
|  | *Spergula* spp. | 0 | 75 | 0 | 75 |
|  | *Taraxacum* spp. | 6 937 | 892 | 386 | 8 215 |
|  | *Tragopogon* spp. | 0 | 0 | 6 | 6 |
|  | *Trifolium repens* L. | 0 | 17 | 78 | 95 |
|  | *Veronica* spp. | 205 | 64 | 29 | 298 |
|  | *Vicia* spp. | 5 | 46 | 491 | 542 |
| **Total** | Total sown | 305 | 3 645 | 12 156 | 16 106 |
|  | Total spontaneous | 8 645 | 2 914 | 2 529 | 14 088 |
|  | Total all flowers | 8 950 | 6 559 | 14 685 | 30 194 |

**Table S3:** Description of each landscape category, with the list of landscape elements included in these categories

| **Landscape categories** | **Description** | **Elements included** |
| --- | --- | --- |
| Semi-natural habitat | Surface of vegetated non-crop habitats that may provide resources, nesting sites and mating sites for pollinators | Forests  Hedgerows  Grasslands  Pastures  Field edges  Road edges  Private and community gardens  Aquatic environments |
| Artificial area | Surface of concrete-covered, gravel and anthropogenic sandy areas. Also includes dwellings. | Houses and buildings  Greenhouses  Concrete and sandy roads  Gravel areas  Cemeteries  Excavation sites |
| Flowering crop | Surface of flowering crops in bloom during our survey | Legume and protein fields  Fruit trees orchards  Blackcurrant orchards  Currants orchards  Rapeseed fields |
| Non-flowering crop | Surface of non-flowering crops and crops not in bloom during our survey. Includes field areas with tilled soil. | Cereal fields  Maize fields  Vineyards |

**Table S4:** List of pollinators observed in blackcurrant orchards. Each individual was identified to the lowest possible taxonomic level and assigned to a morphogroup. Pollinator abundance corresponds to the total number of individuals recorded for each species or genus throughout the surveys, and “n” represents the total number of individuals within each morphogroup.

| **Morphogroup identification** | **Taxonomic identification** | **Pollinator abundance** |
| --- | --- | --- |
| *Apis mellifera*  (n = 408) | *Apis mellifera* Linnaeus, 1758 | 408 |
| *Bombus* spp.  (n = 251) | *Bombus vestalis* (Geoffroy, 1785) | 1 |
|  | Subgenus *Melanobombus* | 35 |
|  | Subgenus *Thoracobombus* | 1 |
|  | Subgenus *Bombus* | 214 |
| Bee fly  (n = 1) | *Bombylius* sp. | 1 |
| Butterfly  (n = 38) | *Lepidoptera* spp. | 38 |
| Hoverfly  (n = 352) | *Syrphidae* spp. | 352 |
| Large wild bee  (n = 270) | *Andrena cineraria* (Linnaeus, 1758) | 7 |
|  | *Andrena flavipes* Panzer, 1799 | 14 |
|  | *Andrena fulva* (Muller, 1766) | 7 |
|  | *Andrena* spp. | 54 |
|  | *Anthophora plumipes* (Pallas, 1772) | 7 |
|  | *Eucera* sp. | 1 |
|  | *Colletes* sp. | 1 |
|  | *Halictus scabiosae* (Rossi, 1790) | 5 |
|  | *Halictus* spp. | 4 |
|  | Other large wild bees | 170 |
| *Osmia bicornis*  (n = 71) | *Osmia bicornis* (Linnaeus, 1758) | 71 |
| Kleptoparasitic bee  (n = 7) | *Nomada* spp. | 5 |
|  | *Sphecodes* spp. | 2 |
| Small wild bee  (n = 606) | *Andrena* spp. | 109 |
|  | *Halictus* spp. | 6 |
|  | *Lasioglossum* spp. | 7 |
|  | Other small wild bees | 484 |
| Total | Total | 2,004 |

**Table S5:** Generalised linear mixed-effects models showing the effects of pollinator and flower abundance in inter-row spaces (inter-row transects), as well as spatial and temporal variables (Table S1), on pollinator abundance in blackcurrant bushes (blackcurrant transects) for each morphogroup: *Bombus* spp., large wild bees, small wild bees, and hoverflies. “n” indicates the total number of pollinators counted. Estimates, Standard Errors (S.E.) and *Z* statistics are indicated. Spatial models were performed for small wild bees, large wild bees, and hoverflies due to spatial autocorrelation in residuals.

*P* < 0.05*, *P* < 0.01**, *P* < 0.001***.

| **Response variable** | **Predictor variables** | **Estimates (± S.E.)** | ***Z*** | ***P*** | **R²m** | **R²c** |
| --- | --- | --- | --- | --- | --- | --- |
| *Bombus* spp. abundance in blackcurrant transects  (n = 118) | Intercept | -0.87 (± 0.13) | -6.77 | < 0.001 *** | 0.01 | 0.02 |
|  | *Bombus* spp. abundance in inter-row transects | 0.28 (± 0.08) | 3.39 | < 0.001 *** |  |  |
| Large wild bee abundance in blackcurrant transects  (n = 120) | Intercept | -1.31 (± 0.36) | -3.66 | < 0.001 *** | 0.06 | 0.15 |
|  | Large wild bee abundance in inter-row transects | 0.39 (± 0.08) | 4.69 | < 0.001 *** |  |  |
|  | Edge - Inner | -0.54 (± 0.22) | -2.49 | 0.013 * |  |  |
|  | Edge - Centre | -0.20 (± 0.30) | -0.68 | 0.499 |  |  |
|  | Round 1 – Round 2 | 0.53 (± 0.25) | 2.13 | 0.033 * |  |  |
|  | Round 1 – Round 3 | 0.68 (± 0.25) | 2.75 | 0.006 ** |  |  |
|  | Semi-natural habitat surface (100m) | -0.36 (± 0.17) | -2.10 | 0.035 * |  |  |
| Small wild bee abundance in blackcurrant transects  (n = 308) | Intercept | -0.86 (± 0.36) | -2.40 | 0.016 * | 0.09 | 0.21 |
|  | Small wild bee abundance in inter-row transects | 0.10 (± 0.05) | 1.85 | 0.065 |  |  |
|  | Edge - Inner | -0.08 (± 0.15) | -0.58 | 0.563 |  |  |
|  | Edge - Centre | -0.71 (± 0.25) | -2.83 | 0.005 ** |  |  |
|  | Round 1 – Round 2 | 0.07 (± 0.15) | 0.49 | 0.627 |  |  |
|  | Round 1 – Round 3 | 0.45 (± 0.14) | 3.16 | 0.002 ** |  |  |
|  | Non-flowering crop surface (100m) | 0.11 (± 0.09) | 1.22 | 0.223 |  |  |
| Hoverfly abundance in blackcurrant transects  (n = 142) | Intercept | -1.94 (± 0.33) | -5.92 | < 0.001 *** | 0.13 | 0.16 |
|  | Hoverfly abundance in inter-row transects | 0.20 (± 0.06) | 3.35 | < 0.001 *** |  |  |
|  | Flowering crop surface (100m) | -0.17 (± 0.09) | -1.81 | 0.070 |  |  |
|  | Round 1 – Round 2 | 1.23 (± 0.32) | 3.91 | < 0.001 *** |  |  |
|  | Round 1 – Round 3 | 1.60 (± 0.31) | 5.12 | < 0.001 *** |  |  |
